# Infratentorial choroid plexus tumors in children

**DOI:** 10.1007/s00381-020-04532-7

**Published:** 2020-03-02

**Authors:** S. Joy Trybula, Constantine Karras, Robin M. Bowman, Tord D. Alden, Arthur J. DiPatri, Tadanori Tomita

**Affiliations:** Division of Pediatric Neurosurgery, Ann & Robert H. Lurie Children’s Hospital of Chicago, Northwestern University Feinberg School of Medicine, 225 E. Chicago Avenue, Chicago, IL USA

**Keywords:** Choroid plexus tumor, Fourth ventricle, Cerebellopontine angle, Pediatric, Posterior fossa

## Abstract

**Objective:**

Choroid plexus tumors (CPTs) are rare pediatric intracranial neoplasms, and mostly occur in the lateral ventricle. CPTs located in the infratentorial location are considered to be rare in the pediatric population. We present a series of eight patients treated in the last decade at our institution focusing on clinical presentations and their outcome after excision.

**Methods:**

We performed an institutional retrospective review of patients who underwent surgical resection of infratentorial CPTs during the period from 2008 to 2017. Patients’ charts were reviewed for demographic data, clinical presentation, surgical treatment, and follow-up.

**Results:**

There were eight patients (6 females and 2 males), with mean age for the cohort at presentation was 9.0 years. They represent 75% of 12 CPTs of all locations treated at the same period in our institution. These 8 infratentorial CPTs were in the fourth ventricle in seven, and in the cerebellopontine angle (CPA) in one. Seven patients had choroid plexus papillomas (WHO grade I) and 1 had an atypical choroid plexus papilloma (WHO grade II). Gross total resection was attempted in all patients. However, two of 3 patients with fourth ventricle floor invasion had subtotal resection with a thin layer of tumor left on the floor. The remaining 6 had a gross total resection. Six patients with preoperative hydrocephalus had a perioperative external ventricular drainage but none required permanent shunting after tumor resection. None showed recurrence/tumor progression without adjuvant therapy during the follow-up period of 20 months to 11 years.

**Conclusion:**

Infratentorial dominance among pediatric CPTs in this series contradicts previous reports. Infratentorial CPTs are amenable to surgical resection. Unresected small residuals due to invasion to the fourth ventricle floor showed no regrowth during 2 to 3 years follow-up without adjuvant therapy. However, these patients with incomplete resection need watchful observations.

## Introduction

Choroid plexus tumors (CPTs) are rare intraventricular tumors of neuroectodermal origin. CPTs occur predominantly in the lateral ventricle in children in contrast with adults who have primarily infratentorial lesions [1, 2]. Choroid plexus tumors are classified by the World Health Organization (WHO) as choroid plexus papillomas (CPP) (WHO grade 1), atypical choroid plexus papillomas (aCPP) (WHO grade 2), and choroid plexus carcinomas (CPC) (WHO grade 3) [3]. Choroid plexus tumors have a predilection for younger age and typically affect patients less than 3 years of age [4, 5]. The survival of choroid plexus papillomas (CPP) is near 100% with gross total resection whereas choroid plexus carcinomas have a poorer prognosis thought to be due to increased local invasion [6]. Many patients present with hydrocephalus and require cerebral spinal fluid (CSF) diversion as a component of their surgical resection. There are few studies and reports of pediatric patients with infratentorial CPTs and particularly those with CPT in the cerebellopontine angle (CPA). There have been less than 10 reported pediatric cases of CPT in the CPA to our knowledge [7–10]. In this study, we present our institutional data of 8 infratentorial tumors surgically treated at our institution over the past decade along with a detailed case report of two selected patients.

## Materials and methods

From 2008 to 2017, eight pediatric patients (aged 23 months–17 years) were treated for histologically verified infratentorial CPTs at Lurie Children’s Hospital in Chicago. All patients underwent tumor resection with an external ventricular drain (EVD) perioperatively. The goal of the surgery was gross total resection with preservation of optimal neurologic outcome. All patients had pre- and postoperative CT and magnetic resonance imaging (MRI) with/without contrast. Histologic diagnosis was assessed according to the WHO grading criteria for CPT. Atypical CPP was diagnosed by histopathological features of the CPP with increased mitotic activity. Data was collected by a retrospective review of patient medical records and was approved by the Institutional Review Board at Lurie Children’s Hospital.

## Results

This study involved 8 patients, 2 males and 6 females. They represent 75% of 12 CPTs of all locations treated at the same period in our institution. Patient characteristics and clinical summaries are given in Table 1. Mean age at presentation for the entire cohort was 9.0 years (range 1.91–17.8 years). Youngest two patients were diagnosed by the age of 23 and 24 months. Common presenting symptoms included signs of increased intracranial pressure such as headaches, emesis, ataxic gait, blurred vision, and dizziness. Three patients became symptomatic following head trauma and subsequent neuroimaging incidentally disclosed the lesion. Duration of symptoms before diagnosis ranged from 1 day to 12 months (median 1 month).

**Table 1:** Patient characteristics and clinical summaries

Case	Sex	Age	Symptoms	Location	Floor invasion	Histology	Extent of resection	HCP	EVD	Shunt	Follow-up
1	M	24 months	Incidental; trauma	4th ventricle	−	CPP	Total	Yes	Yes	No	11 years and 11 months
2	F	17 years and 2 months	Dizziness, headaches, ataxia	4th ventricle	−	CPP	Total	Yes	Yes	No	11 years and 8 months
3	F	17 years 10 months	Incidental; trauma	4th ventricle	−	CPP	Total	No	No	No	1 year and 8 months
4	F	23 months	Ataxic gait	4th ventricle	−	CPP	Total	Yes	Yes	No	9 years and 4 months
5	F	5 years and 7 months	Ataxic gait	4th ventricle	+	CPP	Total	Yes	Yes	No	9 years and 4 months
6	F	17 years and 3 months	Backache, blurred vision	4th ventricle	+	CPP	Subtotal	Yes	Yes	No	3 years
7	F	5 years and 3 months	Emesis, ataxia, headaches	4th ventricle	+	aCPP	Subtotal	Yes	Yes + ETV	No	2 years
8	M	5 years and 2 months	Incidental; trauma	Right CPA	−	CPP	Total	No	No	No	1 year and 9 months

*HCP*, hydrocephalus; *EVD*, external ventricular drainage; *ETV*, endoscopic third ventriculostomy

**Fig. 1:**
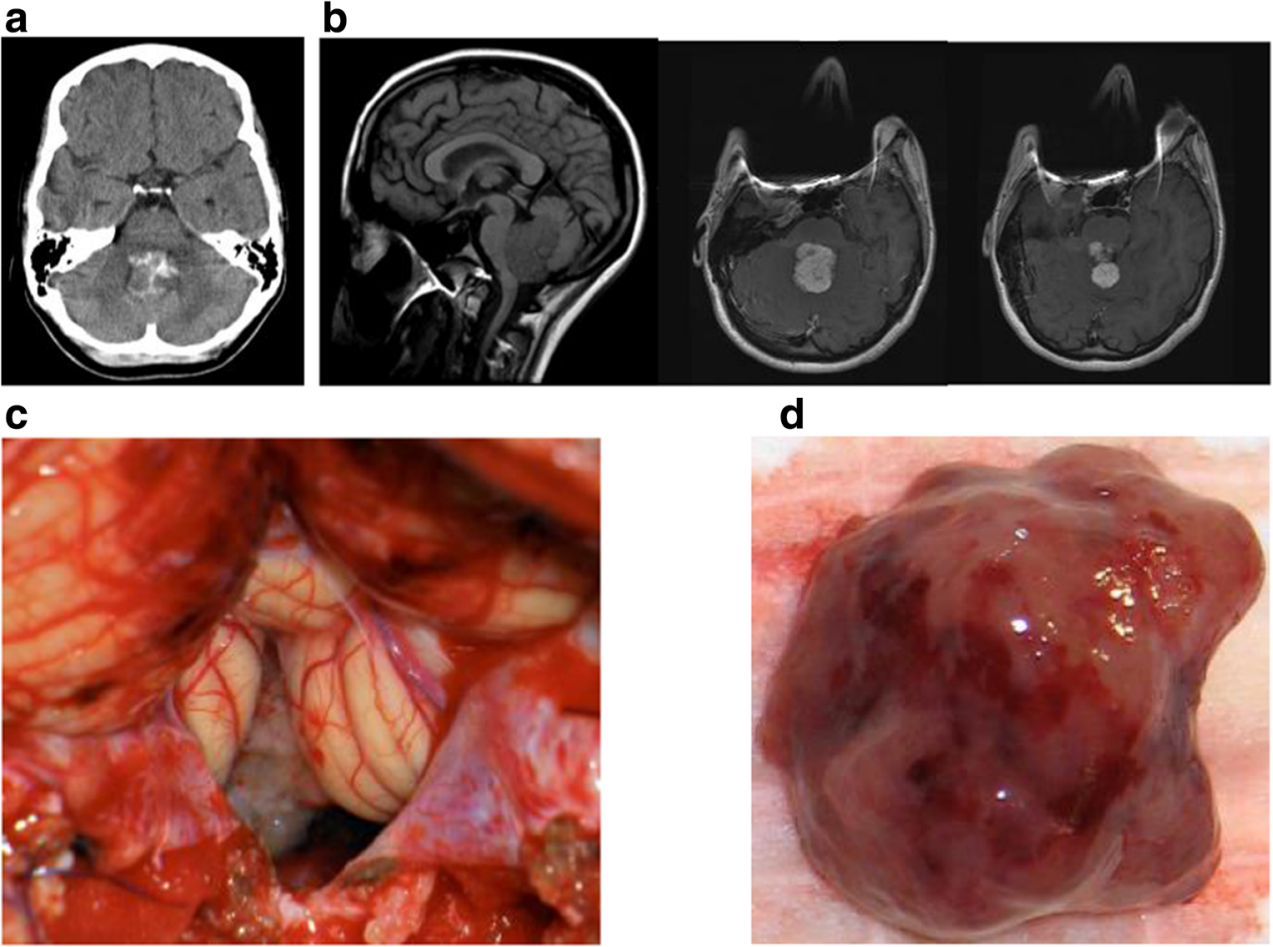
Case 2. **a** Non-contrast enhanced CT showing partially calcified choroid plexus papilloma in the fourth ventricle. **b** Non-contrast enhanced MR T1-weighted sagittal image (left) and contrast enhanced MR T1-weighted axial images (center and right) showing a lobulated enhanced tumor in the fourth ventricle. **c** A surgical photograph showing a forth ventricle tumor through the cerebellar vallecula. **d** Gross surgical specimen of fourth ventricle papilloma. Note edematous salmon roe-like appearance

Six patients had signs of hydrocephalus on imaging. On pre-contrast CT, the lesions were heterogenous, lobulated, and hyperdense relative to the surrounding brain. Various degrees of calcification were observed in four patients (33%) (Fig. 1a). MR imaging demonstrated a T1 and T2 isointense lesion with homogenous contrast enhancement, often lesions were spiculated or lobulated (Fig. 1b).

The primary tumor site was the fourth ventricle in 7 patients, right cerebellopontine angle in 1 patient. Histologically, 7 patients had CPP (WHO grade 1) and 1 patient had an atypical CPP (WHO grade 2). No patients had choroid plexus carcinoma (CPC) in this series.

All patients underwent surgical resection through a posterior fossa craniotomy (Fig. 1c). On surgical observation, the surface of the tumor was homogeneous, frond-like, and relatively avascular, and some showed edematous salmon roe-like appearance (Fig. 1d). The central core of the tumor was more dense and fibrous. Various vascularities were noted, primarily supplied by the posterior inferior cerebellar artery (PICA) and its branches. Three of 8 patients had a tumor invasive to the floor of the fourth ventricle. Total tumor resection was achieved in 6 patients. In two, a subtotal resection was completed leaving a thin sheath of tumor over the floor (cases 6 and 7), while another (case 5) had a gross total resection. No blood transfusions were required for these cases intraoperatively or postoperatively.

Six patients with hydrocephalus had EVD placement prior to the craniotomy under the same anesthesia. One of them underwent an endoscopic third ventriculostomy (ETV) at the same time of EVD (case 7). All EVDs were successfully removed following tumor resection prior to patient discharge. No patients required ventricular shunt placement postoperatively. Of our cohort, all are known to be alive without tumor progression during the mean follow-up of 6.3 years (range 20 months–11.9 years). At the last follow-up, all patients were neurologically normal except for one (case 7) with facial and ocular palsy.

## Illustrative cases

### Case 7

**Fig. 2:**
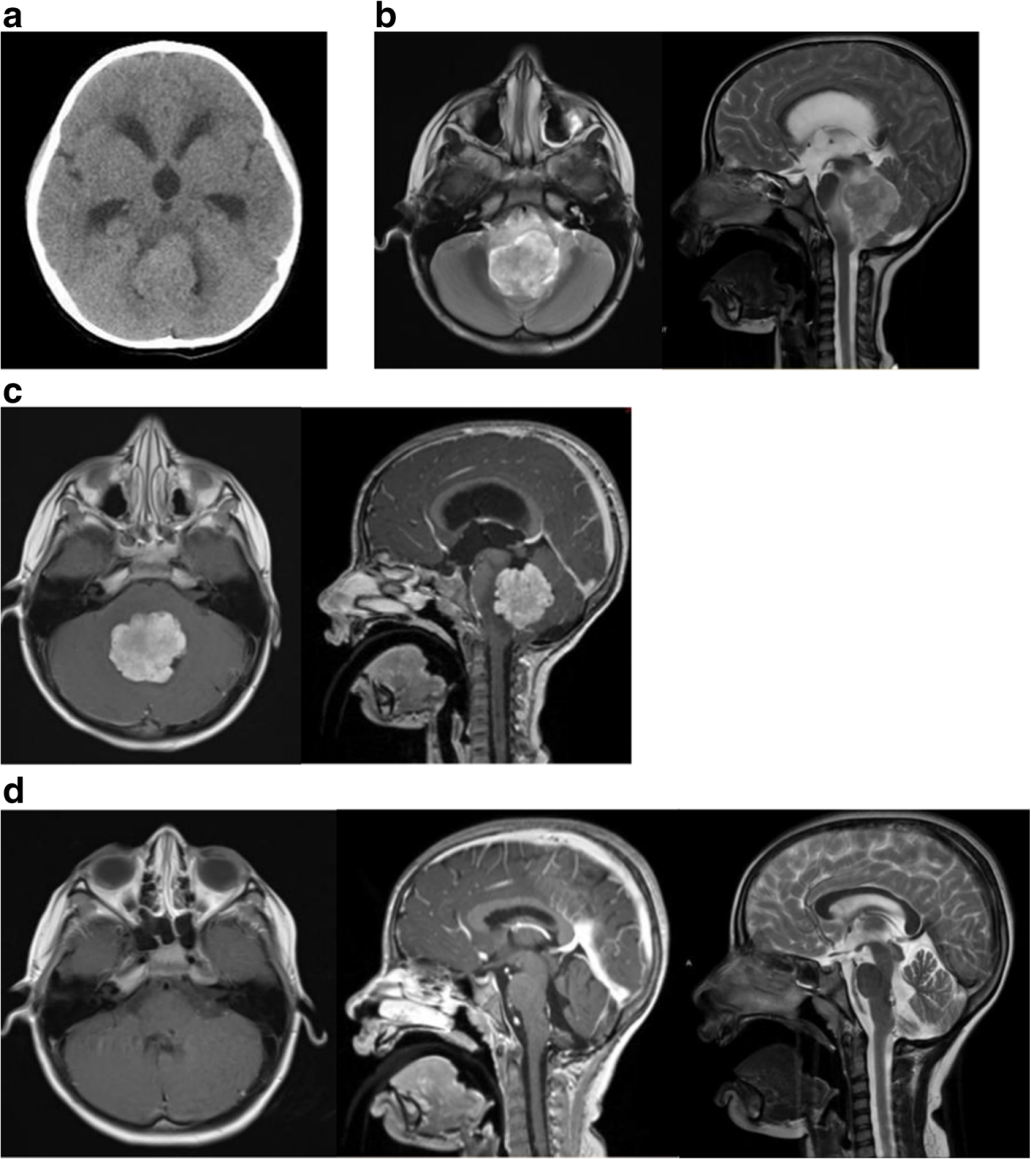
Case 7. **a** Non-contrast enhanced CT showing an isodense lesion centered in the fourth ventricle with moderate ventriculomegaly. **b** T2-weighted MR, axial image (left) and sagittal image (right) showing a severe compression on the left-sided floor of the IV ventricle and severe hyperintense changes in the dorsal brainstem and cerebellar peduncle by the IV ventricle tumor. **c** Contrast enhanced MRI, T1-weighted axial image (left) and sagittal image (right) showing a lobulated homogenously enhancing tumor centered in the IV ventricle. **d** Postoperative MRI brain; contrast enhanced T1-weighted axial image (left) and sagittal image (center) showing a resolution of IV ventricle tumor with a small residual enhancement on the floor of the IV ventricle, and resolution of brainstem edema on T2-weighted sagittal image (right)

A 5-year-old female presented with 8 months of daily nausea/emesis, loss of body weight, and occasional bifrontal headaches. She began to show increasing unsteady gait for 1 month. Neurological examination showed inability to gaze towards her left side, mild left side esotropia and facial droop and profound truncal ataxia. CT (Fig. 2a) and MR showed a lobular mass in the IV ventricle compressing the left-sided pons causing severe T2 hyperintensity in the dorsal brainstem and cerebellar peduncles (Fig. 2b). MRI with contrast showed a mass with lobulated frond-like margins and intense nearly homogenous enhancement (Fig. 2c).

She underwent ETV and EVD for hydrocephalus and then under the same anesthesia, a posterior fossa craniotomy for tumor resection. There were multiple feeding branches noted from the bilateral PICAs, with more feeders noted from the left-sided PICA. The central core of the tumor was densely fibrous and vascular. The floor of fourth ventricle was invaded more to the left side at the pons. The tumor was excised in a piecemeal fashion except for the small portion of tumor invading the floor at the level of the lower pons of the left side.

Pathology showed a choroid plexus papilloma without nuclear pleomorphism but with increased mitotic activity (3 mitoses/10HPF) and an increased Ki-67 proliferation index (5% overall, focally up to 15%). The tumor had these findings were consistent with an aCPP.

#### Postoperative course

Immediate postoperative course was complicated by transient posterior fossa mutism, which completely resolved 4 weeks after the operation. Her cerebellar ataxia also resolved around the same time. However, she had persistent left facial weakness House-Brackmann (HB) stage V/VI, and partial bilateral internuclear ophthalmoplegia. She had a cross-facial nerve animation procedure for persistent left facial weakness 6 months after the operation. At the last follow-up, 24 months after the surgery, her facial paralysis improved to HB stage III. She had persistent partial internuclear ophthalmoplegia without double visions. Follow-up MR shows a stable small lesion on the floor of the fourth ventricle while hyper intense lesion in the brainstem resolved (Fig. 2d).

### Case 8

**Fig. 3:**
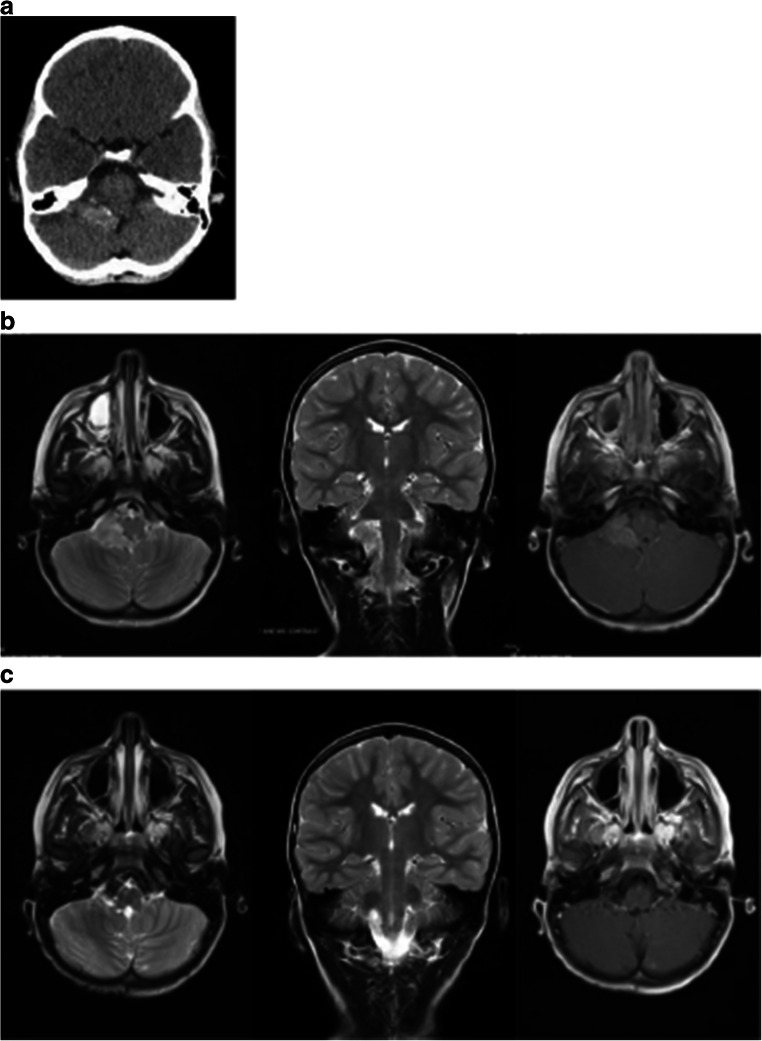
Case 8. **a** Non-contrast CT showing a hyperdense right cerebellopontine angle lesion centered around the foramen of Luschka. **b** Preoperative T2-weighted MR; axial (left) and coronal (center) images and contrast enhanced T1-weighted axial image (right) show homogeneous enhancement of a CPA choroid plexus papilloma. **c** Postoperative T2-weighted MR; axial (left) and coronal (center) images and T1 with contrast (right) show a resolution of a CPA choroid plexus papilloma

A 5-year-old male had a minor head-on collision followed by headaches. CT without contrast obtained at that time showed a lobulated hyperdense mass at the right CPA (Fig. 3a). Subsequent MRI showed a lobulated mass, hyperintense on T2, and homogeneously enhancing right CPA lesion (Fig. 3b). There were no signs of hydrocephalus. His neurological examination was intact.

He underwent a posterior fossa craniotomy tumor resection. It was done through a hockey stick incision as outlined previously [11]. A homogeneous solid tumor was present in the right cerebellomedullary fissure of the CPA. The nerve roots of 7–10th cranial nerves of the right side were displaced ventrally. Medially the right-sided PICA gave rise multiple feeders to the tumor. The tumor appeared to be present in the expanded lateral recess with the tela choroidea surrounding it dorsally. There was no brainstem involvement. A piecemeal resection was done under surgical microscope, and a gross total resection was achieved. Pathology was consistent with CPP.

The patient showed no postoperative neurological deficits. Presently, 21 months after the tumor resection, he shows no signs of tumor recurrence (Fig. 3c).

## Discussion

CPTs in the pediatric population are typically discovered within the first decade of life and particularly within the first 2 years of life [12, 13]. Patients with infratentorial choroid plexus tumors are older at presentation compared with those patients with supratentorial tumors, which is consistent with previous literature [4, 5, 13]. In our cohort, the mean age at detection of infratentorial lesions is 9.0 years. We encountered a higher rate of infratentorial CPT location (75%) in our patient population over the last decade, which is contrary to the rates reported in prior studies and our own prior experiences [10, 14, 15]. We did not have any CPC in our cohort; however, the incidence of CPC among CPTs of all locations is approximately 11% in prior studies [2], and its infratentorial occurrence is exceedingly uncommon [16]. However, others reported anaplastic choroid plexus carcinoma is a tumor with a predilection for the posterior fossa of infants [17].

CPTs arise from the choroid plexus. The choroid plexus of the fourth ventricle consists of two symmetrical parts located in the roof of the ventricle and protruding through the foramina of Luschka and Magendie. PICA supplies most of the choroid plexus in the roof and the median opening of the fourth ventricle, whereas anterior inferior cerebellar artery (AICA) supplies the portion of the plexus in the CPAs and the adjacent part of the lateral recess [18]. All our patients including the one of CPA tumor, however, had vascular supply from PICA.

The CPA is a rare site for CPT during childhood. Luo et al. reviewed all CPTs of CPA in both the adult and pediatric population and of the 21 patients, they had only 2 pediatric patients (9%) [10]. Cranial nerve preservation at the CPA during the tumor resection is essential to avoid postoperative complications. Most of our patients presented with headache and gait ataxia while some were otherwise asymptomatic. Visual symptoms such as abducens palsy or inability to lateral gaze are due to hydrocephalus or direct brainstem compression/invasion, respectively. Patient 7 with tumor invasion to the floor of the fourth ventricle presented with more severe symptoms of ataxic gait and ocular disturbance due to PPRF and facial colliculus who presented with direct brain stem compression and edema. An invasive CPP to the lateral floor near the lateral recess of the left side of case 5 was successfully removed. He had only left-sided dysmetria but without signs of residual or recurrence for more than 9 years. Invasive tumor to the floor of the 4th ventricle should be resected conservatively. Even with small residual tumor on the ventricular floor, there has been no recurrence without adjuvant therapy to date in our patients 6 and 7, for 3 and 2 years, respectively.

The current standard of care for CPT is surgical gross total resection [12, 19, 20]. These lesions are known to be highly vascular and early control of the arterial feeding vessels is important to maintain hemostasis. It is common especially in the pediatric population to require blood transfusion given the lower total circulating blood volume [21, 22]. However, we noted no significant blood loss during the resection of infratentorial CPPs in this series. None required blood transfusion or preoperative embolization for the posterior fossa lesions. Our radiographic findings were similar to prior studies particularly with the spiculated, calcification, contrast enhancement, and frond-like projections [13, 23]. These frond-like tissues are relatively avascular and reduced in volume by bipolar cautery and suction.

## Conclusion

None of the 6 patients in our cohort had recurrence or progression on imaging after total resection. In 2 patients, following incomplete resection due to brainstem involvements, no recurrence was noted during 2 and 3 years follow-up time. Inability of total resection of CPP did not necessarily increase risk of recurrence based on our relatively short follow-up in our cohort. Continuous surveillance follow-up is recommended.
